# A matter of background: DNA repair pathways as a possible cause for the sparse distribution of CRISPR-Cas systems in bacteria

**DOI:** 10.1098/rstb.2018.0088

**Published:** 2019-03-25

**Authors:** Aude Bernheim, David Bikard, Marie Touchon, Eduardo P. C. Rocha

**Affiliations:** 1 Microbial Evolutionary Genomics, Institut Pasteur, CNRS, UMR3525, 25-28, rue Dr Roux, Paris, 75015, France; 2 Synthetic Biology Group, Institut Pasteur, 25-28 rue Dr Roux, Paris 75015, France; 3 AgroParisTech, Paris 75005, France

**Keywords:** CRISPR-Cas systems, horizontal gene transfer, epistasis, immunity, recombination

## Abstract

The absence of CRISPR-Cas systems in more than half of the sequenced bacterial genomes is intriguing, because their role in adaptive immunity and their frequent transfer between species should have made them almost ubiquitous, as is the case in Archaea. Here, we investigate the possibility that the success of CRISPR-Cas acquisition by horizontal gene transfer is affected by the interactions of these systems with the host genetic background and especially with components of double-strand break repair systems (DSB-RS). We first described the distribution of systems specialized in the repair of double-strand breaks in Bacteria: homologous recombination and non-homologous end joining. This allowed us to show that such systems are more often positively or negatively correlated with the frequency of CRISPR-Cas systems than random genes of similar frequency. The detailed analysis of these co-occurrence patterns shows that our method identifies previously known cases of mechanistic interactions between these systems. It also reveals other positive and negative patterns of co-occurrence between DSB-RS and CRISPR-Cas systems. Notably, it shows that the patterns of distribution of CRISPR-Cas systems in Proteobacteria are strongly dependent on the epistatic groups including RecBCD and AddAB. Our results suggest that the genetic background plays an important role in the success of adaptive immunity in different bacterial clades and provide insights to guide further experimental research on the interactions between CRISPR-Cas and DSB-RS.

This article is part of a discussion meeting issue ‘The ecology and evolution of prokaryotic CRISPR-Cas adaptive immune systems’.

## 1. Introduction

CRISPR-Cas are adaptive immune systems that protect Bacteria and Archaea from phages and other mobile genetic elements (MGEs). They are composed of a CRISPR array (clustered regularly interspaced spacer with palindromic repeats) and a cluster of 'Cas' genes (CRISPR-associated genes). CRISPR-Cas immunity works in three stages: adaptation, generation of CRISPR RNAs (crRNAs) and immunity [1]. During adaptation, the system takes in new spacers from foreign genetic elements and integrates them in the CRISPR array. During immunity, the CRISPR array is transcribed, processed and used by a complex of Cas proteins for sequence-specific recognition and subsequent cleavage of foreign DNA [1]. Given the efficiency of CRISPR-Cas systems and their high rate of horizontal gene transfer between lineages [2], their absence from the majority of bacterial genomes remains a puzzle [3,4]. As a point of comparison, there are, on average, two restriction-modifications systems per bacterial genome [5]. These observations are also intriguing in the light of near ubiquity of CRISPR-Cas systems in archaea. Several hypotheses have been put forward to explain this observation, but they are not completely satisfactory. The acquisition of a self-targeting spacer leads to autoimmunity which in the majority of cases results in cell death [6]. However, it is unclear why the cost of autoimmunity should vary between clades. One might consider that innate defences, like restriction modification or surface modification, can be more advantageous than encoding a specialized defence system like CRISPR-Cas, depending on the risk of infection and the cost of immunity [7]. But this does not explain why many environments have bacteria with and bacteria without CRISPR-Cas systems. Finally, CRISPR-Cas systems prevent the uptake of MGEs such as plasmids [8] that may carry advantageous traits [2]. However, this is also true for the other defence systems, and recent work suggests that the efficiency of transduction could be increased by the presence of CRISPR-Cas targeting transducing phages [9]. In this case, CRISPR-Cas might actually favour allelic recombination within species. All these costs of CRISPR-Cas systems seem to affect Bacteria and Archaea, but the frequency of these systems is dramatically different in the two clades.

Here, we propose that successful acquisition of a CRISPR-Cas system depends on the genetic background, and in particular on the repertoire of functions associated with DNA double-strand break (DSB) repair systems (DSB-RS, reviewed in [10–12]), which are very different between Archaea and Bacteria. This is because CRISPR-Cas systems produce single-stranded or DSBs and can complement or compete with housekeeping functions to deal with such lesions. Accordingly, there is increasing evidence for direct interaction between the two types of systems [13].

There are three major pathways of DSB-RS in bacteria. Most species repair DSBs using the pre-synaptic pathways AddAB, RecBCD or AdnAB [10,14,15] involved in repair by homologous recombination. These are protein complexes including different combinations of helicase and nuclease domains that recognize, process and load RecA on DSBs [12]. In certain genetic backgrounds, in particular when RecBCD and other exonucleases like SbcB and SbcCD are absent, the RecFOR homologous recombination pathway processes DSB and loads RecA. This pathway is also the one implicated in managing single-stranded breaks in DNA, which can give rise to DSBs upon replication [10]. The RecFOR pathway also includes the helicase RecQ and the nuclease RecJ [10]. The role of RecN during DNA repair is to promote contacts between sister chromatids. It modulates whole chromosome organization and RecA dynamics [16]. Strand exchange in bacterial homologous recombination is usually catalysed by RecA, a multifunctional protein also involved in the regulation of the SOS response [17]. The DNA molecules joined by the action of RecA are then resolved by the RuvABC complex (RuvAB and RecU in some Firmicutes) [10]. The role of RecG in homologous recombination, once thought to be complementary to that of RuvABC, is still subject to controversy. It prevents over-replication and the processing of R-loops [18,19]. This list of key proteins involved in DSB-RS is accompanied by many others that are associated with recombination, but have either poorly defined or very pleiotropic functions. In this study, we analysed the following: SbcEF because it has been implicated in DSB repair, even if its precise role remains poorly characterized [20]; RecX because it is a modulator of the activity of RecA [21]. Finally, LexA because it is activated by RecA leading to the SOS response following detection of DSB in the cell [22]. A third mechanism involved in DSB-RS, and analysed in this study, is non-homologous end joining (NHEJ), which requires the DNA-end binding protein Ku and a ligase to repair DSB without a template [11].

There is evidence that some of these proteins are necessary for the correct function of CRISPR-Cas systems. For example, the adaptation step in the subtype I-E system depends on the integration host factor [23,24] and is favoured by the helicase activity of RecBCD enzyme [25,26]. Other proteins associated with DSB-RS are involved in CRISPR-Cas adaptation [27,28]. All these molecular mechanisms act on DNA and can compete for the same substrate. This can result in a mechanism physically blocking the access to DNA or reverting the action of another. Accordingly, CRISPR-Cas subtype II-A was recently shown to affect the ability of NHEJ to repair DSB [29]. DSBs produced by CRISPR-Cas systems can also be repaired by NHEJ, as observed in Eukaryotes and Bacteria [30,31], although it is unclear whether this can affect the efficiency of CRISPR immunity [29].

In this study, we start by assessing the distribution of DSB-RS, because this was last done over a decade ago [32]. Then we examined the pattern of co-occurrences of DSB-RS and CRISPR-Cas systems in bacterial genomes to identify positive and negative associations. Systems interacting synergistically with a specific repair pathway are expected to co-occur more often than expected by chance. Inversely, negative interactions are expected to lead to less co-occurrence than expected.

## 2. Material and methods

### (a) Data

We analysed 5563 complete genomes retrieved from NCBI RefSeq (ftp://ftp.ncbi.nih.gov/genomes/, last accessed in November 2016) representing 2437 species of Bacteria.

### (b) Detection of CRISPR-Cas systems

CRISPR-Cas systems were detected with CasFinder v. 2.0 [33]. CasFinder exploits MacSyFinder (v. 1.0) [34], a program that uses protein profiles and a set of rules concerning quorum and organization of components to identify molecular systems in genomes. Briefly, Cas proteins were detected using HMM profiles and systems were then discriminated at the subtype level based on the appropriate models. Three proteins are required to form a Cas system in class I systems, one for class II. The subtype assignment is achieved through signature proteins (Cas9 and Csn2 for subtype II-A, for example). All results are reported in electronic supplementary material, table S1.

### (c) Detection of DNA repair pathways

We used MacSyFinder (v. 1.0.2) [34] to detect the components of DSB-RS. For this, we defined the models—protein profiles and organization rules—to identify these systems. The protein profiles used in these searches were either retrieved from TIGRFAM or built from scratch when no adequate profiles existed (AdnA, AdnB, SbcB, SbcE) or when detection using TIGRFAM profiles missed known homologues (AddA, AddB) (electronic supplementary material, table S2) (see below for details on the building of HMM profiles). We defined genetic organization rules based on the literature [10,32,35,36] (electronic supplementary material, table S2). We compared these results to MacSyFinder analyses using other methods in smaller sets of genomes [32,35]. Default parameters of MacSyFinder were used except in specific cases described in electronic supplementary material, table S2. All HMM profiles and definition are provided in the electronic supplementary material.

### (d) Construction of protein profiles

New protein profiles for the proteins involved in DSB-RS were built using a homogeneous procedure. We collected a set of sequences from the protein family that were representative of the diversity of the bacterial taxonomy (see below for details). The homologous proteins were aligned using MAFFT v. 7.205 (default options, mode auto) [37]. Multiple alignments were manually curated using Seaview v. 4.6.2 [38] and then used to produce protein profiles with hmmbuild (default options) from the HMMer [39] suite v. 3.1.

For AddA and AddB, we first obtained a list of representative proteins from different clades as described in [35]. As known functional homologues in Epsilonproteobacteria were not detected by these customized profiles, two specific profiles to detect AddA and AddB in Epsilonproteobacteria were built using sequences from a previous publication [40]. We compared our results with that of Cromie [35], which had several orders of magnitude fewer genomes, and checked that both works identified AddAB in all genuses analysed in both works (with the exception of Wolbachia, where the hits to the profiles developed in this work were not statistically significant). We sometimes identified AddAB pseudogenes (e.g. in some *Staphylococcus*, *Burkholderia* or *Bordetella*), in which case the system was indicated as absent.

For AdnA, AdnB, SbcB and SbcE, we used curated proteins from Uniprot as a starting point and used Blast (Blast-p -> NCBI, May 2016) to fetch homologues from the non-redundant protein sequences database of NCBI. All hits belonging to different clades among the 250 best hits with more than 40% identity were selected and aligned as described above.

### (e) Persistent genomes of Firmicutes and Proteobacteria

We inferred the families of orthologous proteins for a set of 1189 genomes of Firmicutes and a set of 2897 genomes of Proteobacteria (larger than 1 Mb) available in the GenBank RefSeq dataset, as indicated above. A list of orthologues was identified as reciprocal best hits using end-gap free global alignment, between the proteome of a pivot and each of the other strain's proteomes (as in [41]). We used as a pivot *Escherichia coli* K12 MG1655 for Proteobacteria and *Bacillus subtilis* str.168 for Firmicutes. Hits with less than 37% similarity in amino acid sequence and more than 20% difference in protein length were discarded. The persistent genome of each clade—the list of families of orthologous proteins present in more than 90% of the genomes—was defined as the intersection of pairwise lists of orthologues that were present in at least 90% of the genomes representing 411 families for Firmicutes and 341 for Proteobacteria.

### (f) Phylogenetic trees

We made phylogenetic trees for each clade from the concatenate of the multiple alignments of the persistent proteins obtained with MAFFT v. 7.205 (with default options) and BMGE v. 1.12 (with default options). The missing proteins were replaced by stretches of ‘-’ in each multiple alignment. Adding ‘-’ has little impact in the reconstruction of the phylogeny as long as these are not very numerous [42]. Each clade tree was computed with FastTree v. 2.1 under LG model [43]. In both cases, the LG model had lower AIC than the WAG model. We made 100 bootstraps to assess the robustness of the phylogenetic reconstruction using phylip's SEQBOOT (default parameters, v. 3.697) [44] to generate resampled alignments and the –n–intree1 options of FastTree.

### (g) Genes analysed in Firmicutes and Proteobacteria

The association studies were done with components/subtypes present in more than 1% and less than 99% of the genomes of a clade. The complete lists are indicated below.

#### (i) Double-strand break repair systems components

Firmicutes: AddAB, RecJ, RecQS, RecX, RecU, RuvC, SbcCD, SbcEF, NHEJ, LexA; Proteobacteria: AddAB, RecBCD, RecF, RecOR, RecG, RecJ, RecQS, RecX, RecN, SbcB, SbcCD, NHEJ, LexA.

#### (ii) Cas subtypes

Firmicutes: type IB, type IC, type IE, type IIA, type IIC, type IIIA, type IIIB, type IIIC, type IIID; Proteobacteria: type IB, type IC, type IE, type IF, type IU, type IIB, type IIC, type IIIA, type IIIB, type IIID, type V.

### (h) Statistical analysis of the associations

We built 2 × 2 contingency tables for all possible associations between DSB-RS and CRISPR-Cas subtypes that were present in more than 1% and less than 99% of the studied bacterial genomes (for example, RecA was not analysed as it is present in more than 99% of Proteobacteria and Firmicutes). This information was the basis for two analyses.

First, we assessed if the presence of components DSB-RS was more associated with the presence of Cas systems than random proteins with similar frequency in the genomes of the same phyla. For each DSB-RS component, we randomly selected 10 proteins with a similar frequency in our dataset of the respective clade (allowing for a margin of ±1% in frequency) which we call the *control genes*. We computed 2 × 2 contingency tables for the co-occurrence of DSB-RS and Cas subtypes. We did the same for the co-occurrence of the 10 control genes with Cas subtypes. We then computed the *Φ* association coefficient for each of the 11 contingency tables (test 16 g in [45]):

$$\Phi =\frac{ad-bc}{\sqrt{\left(a+b\right)}(c+d)(a+c))b+d)},$$

Where *a* (row 1, column 1), *b* (row 1, column 2), *c* (row 2, column 1) and *d* (row 2, column 2) are the counts in the contingency table. An association coefficient significantly larger than one indicates frequent co-occurrence, whereas a coefficient significantly negative indicates avoidance (lower than expected co-occurrence). The absolute value of the coefficient indicates the strength of the association, independently of its original sign.

We then calculated the difference (Δ*Φ*) between the absolute value of the *Φ* coefficient of the associations involving the DSB-RS and the Cas subtype and the average of the absolute values of the *Φ* coefficient for the association of the control genes and the Cas subtype. This difference indicates the extent to which one of the two (DSB-RS if positive and control genes if negative) has a larger absolute association with Cas subtypes. We took all the differences—corresponding to all the analysis of DNA repair systems and Cas subtypes—and used a one-sided *t*-test to test if we could reject the null hypothesis H0: the mean of Δ*Φ* is null or negative versus H1: the mean of Δ*Φ* is positive.

Second, we detailed the significance of each individual association of a component of the DSB-RS and a Cas subtype. We used a Fisher exact test to test if the respective association in the 2 × 2 contingency table was significant. Since this resulted in a large number of tests (246 tests), we used a Bonferroni correction to identify the significant ones (*α*-value 0.05). We then tested if these significant associations could be the result of phylogenetic correlation using BayesTraits v. 3.0 [46], where we used as input the table with information on presence or absence of the traits and the phylogenic tree of the clade (Firmicutes or Proteobacteria, see above for the phylogenetic reconstruction). We estimated the likelihood of the presence or absence of the two traits using two models: one where it is hypothesized that the discrete traits evolved independently and one where the characters evolved in a correlated manner. The likelihood-ratio test was used to assess the statistical significance of the more complex model (correlated evolution). To assess the robustness of this test to uncertainties in phylogenetic inference, we computed likelihood-ratio tests on 100 trees inferred from 100 bootstrap alignments (same procedure used for the phylogenetic reconstruction). We considered that an association was significant after phylogenetic correction if the median of those 100 likelihood ratio tests was inferior to 0.01.

### (i) Clustering

We clustered the associations between variables by assigning to each association a value: 0 (non-significant), −1 (significant and negative) or 1 (significant and positive). The matrix of these associations was clustered using hierarchical clustering (clustermap function from the seaborn package in Python 2.7 with default parameters). The function uses the nearest neighbour algorithm method to form the clusters.

## 3. Results and discussion

### (a) Distribution of DNA repair pathways in bacterial genomes

**Figure 1.**
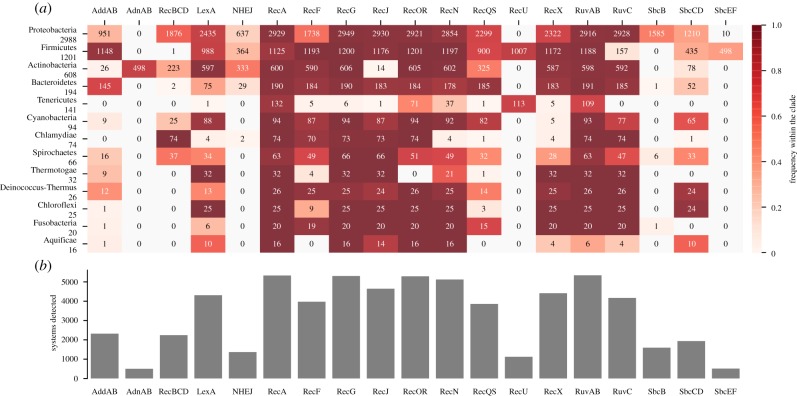
Distribution of DSB-RS in bacterial genomes. (*a*) The distribution of the components (*x*-axis) in the bacterial phyla with most sequenced genomes. Clades are ordered by number of genomes present in the dataset which are indicated on the *y*-axis. The number in the cells represents the number of detected elements and the colour indicates their frequency in the clade. (*b*) The total number of components detected in the dataset.

We detected CRISPR-Cas systems and proteins involved in DNA repair in 5563 fully sequenced bacterial genomes (electronic supplementary material, table S1). Given the lack of recent works describing the frequency of the DSB-RS, we start this report by describing succinctly these data (for CRISPR-Cas, see [3,33]). Several components implicated in DSB-RS are nearly ubiquitous in Bacteria: RecA, the resolvases RuvAB, RecG and the pre-synaptic system RecOR. All of these could be detected in more than 96% of the genomes (figure 1). They represent the nearly ubiquitous toolkit of homologous recombination in Bacteria. Careful inspection of the genomes lacking RecA, the hallmark of the presence of homologous recombination, showed that they were small and usually also lacked the other DSB-RS. We used tfastx v. 36 (*e*-value < 0.01, using *B. subtilis* RecA as a reference [47]) to search for RecA pseudogenes or annotation errors in these genes. Most of the genomes that were missed in the identification of RecA showed vestiges of the gene under the form of pseudogenes, or had group I self-splicing introns that were poorly annotated (*Bacillus cereus* group [48]). Only 20 species, out of 2237, lacked any evidence of the presence of RecA (electronic supplementary material , table S3), all of them with very small genome size (electronic supplementary material, figure S1). These results confirm previous findings that homologous recombination machineries are present in most Bacteria with exception of some small genomes of obligatory symbionts [49].

The distribution of less ubiquitous components of DSB-RS also confirmed analyses done over a decade ago on a much smaller number of genomes [32,35,50]. Some of these systems are not very frequent because they are part of different epistatic groups with similar functions (figure 1). For example, 88% of the genomes encode either RecBCD, AddAB or AdnAB, even if each of them is present in less than half of the genomes. Actually, only 16 genomes encode both RecBCD and AddAB, even if both pathways are very frequent in Proteobacteria. The AdnAB pathway was exclusively found in Actinobacteria, where some genomes also encode RecBCD (but the latter seems to be involved in single-stranded annealing and not in homologous recombination in this phylum) [51]. The resolvases have complementary patterns of occurrence that largely follow the taxonomy: RecU is only found in Fimicutes and Tenericutes, whereas RuvC is found in some of the former and in most genomes of the remaining phyla. Together, they are present in more than 96% of the genomes, matching the frequency of the nearly ubiquitous components described above.

The systems that are not directly implicated in homologous recombination show more diverse distributions. LexA is present in most bacteria, even if its frequency varied with the clade, suggesting that SOS responses are present in most phyla. NHEJ is present in 25% of bacterial genomes and is particularly abundant in Actinobacteria, where 55% of the genomes encode NHEJ. Overall, these results confirm the near ubiquity of the major functions involved in the repair of DSB by homologous recombination and the relative rarity of NHEJ in the bacterial world.

### (b) Interactions between Cas subtypes and double-strand break repair systems in Proteobacteria and Firmicutes

We used the information on the presence of the different components of DSB-RS and CRISPR-Cas systems to test for significant associations between them. For this, we assessed if these associations were more frequent than expected, given the frequency of these systems in genomes. Subsequently, we detailed these results while taking phylogeny into account (see below). We concentrated our efforts on Proteobacteria and Firmicutes because deeper phylogenetic associations are hard to define accurately. These two clades include most (75%) of the available genomes, and accumulate most known information on both DSB-RS and CRISPR-Cas systems. We only analysed components/subtypes present in more than 1% and less than 99% of the genomes of Firmicutes and Proteobacteria. This resulted in a dataset of 10 different DSB-RS components in Firmicutes and 13 in Proteobacteria, as well as nine CRISPR-Cas subtypes in Firmicutes and 11 in Proteobacteria (see Material and methods for the complete lists).

We then investigated whether CRISPR-Cas are more frequently associated with the presence or absence of DSB-RS than with other cellular functions. For the analysis of each DSB-RS component, we selected 10 random genes with other cellular functions that displayed a similar frequency in the genomes of the same phyla (Proteobacteria or Firmicutes). We then computed the coefficient of association of the 11 (10 controls and the DSB-RS) 2 × 2 contingency tables (*Φ*). Note that the *Φ* values between the DSB-RS and the controls were positively correlated because most DSB-RS genes are present in most genomes and thus their control genes are also very frequent in the same genomes. Nevertheless, the *Φ* values for the association of DSB-RS genes with CRISPR-Cas systems were significantly higher than the *Φ* values for the association of control genes with CRISPR-Cas systems in Firmicutes and in Proteobacteria (one-sided *t*-test for their paired difference, *p* = 0.0003 for Firmicutes *p* = 0.0346 for Proteobacteria, electronic supplementary material, figure S3). We thus conclude that CRISPR-Cas systems present more associations with DSB-RS than with proteins with other cellular functions.

**Figure 2.**
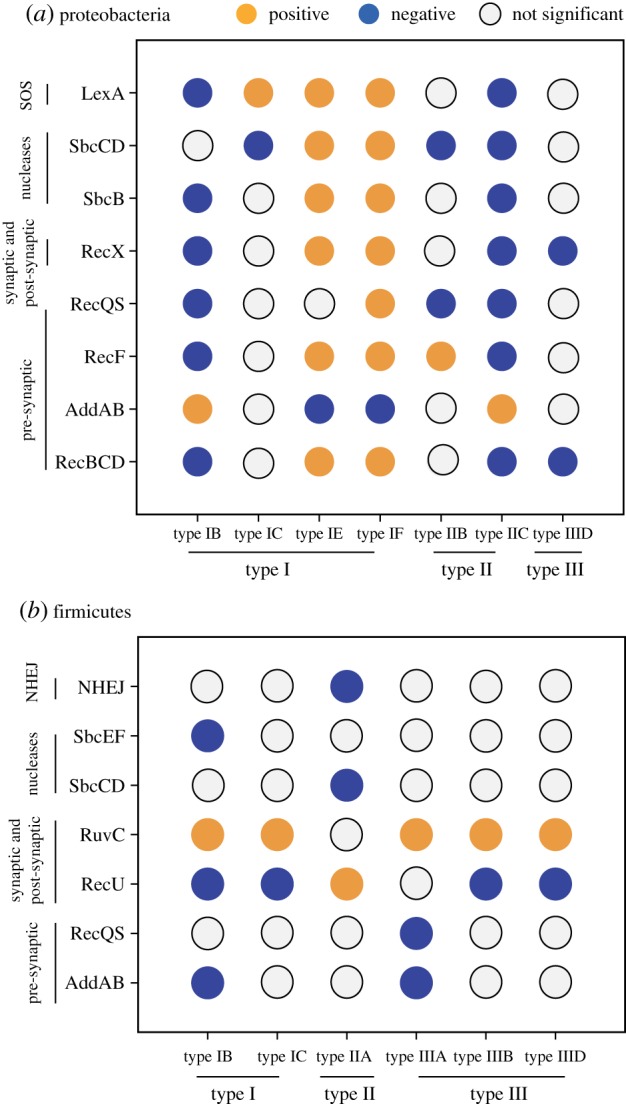
Associations between CRISPR-Cas systems and DSB-RS in Proteobacteria (*a*) and Firmicutes (*b*). Each circle corresponds to the association between a CRISPR-Cas system on the *x*-axis and a DSB-RS component in the *y*-axis. Colour code: no significant association (grey), negative association (blue) and positive association (orange). Association was tested by a Fisher exact test *p* < 0.05 with a Bonferroni correction followed by the phylogenetic dependence test using the median of 100 likelihood ratio tests (if median lower than 0.01). Only systems present in more than 1% and in less than 99% of the total number of genomes in the clade and presenting at least one significant association are represented.

We used the 2 × 2 contingency tables to identify when DSB-RS and Cas subtypes did not occur independently. If the pair failed the independence test (Fisher exact test at *α* = 0.05 with a Bonferroni correction for multiple tests), we made an additional test controlled by the phylogeny [46,52,53]. DSB-RS tend to be conserved at the species level, but CRISPR-Cas systems sometimes vary between strains. Hence, we analysed the patterns of co-occurrence at the genome level, i.e. we analysed all genomes even when they were from the same species. This increased the size of the datasets, at the cost of increasing phylogenetic dependence. To control for the latter, we inferred the phylogenetic trees for the Firmicutes and for the Proteobacteria, and used them to test the co-occurrence of systems with BayesTraits [46]. This revealed 53 positive and negative significant associations out of a total of 233 possible (figure 2).

We first concentrated on testing if previously reported interactions were retrieved by our method. We detected a positive association between the presence of RecBCD and subtype I-E CRISPR-Cas systems in Proteobacteria (RecBCD is lacking in Firmicutes). This association is consistent with the synergistic interaction between both pathways recently experimentally observed in *E. coli* [25,26]. We also observed a negative association between subtype II-A CRISPR-Cas systems and NHEJ in Firmicutes, as indicated and experimentally confirmed in our previous report [29]. Recently, it was reported that RecG contributes to primed adaptation—acquisition of novel spacers from an MGE already targeted by a spacer present in the CRISPR array—in subtype I-E and I-F systems [27,28]. We were unable to detect this interaction in our analysis, presumably because RecG is present in 99% of the genomes larger than 1 Mb and the test lacks statistical power. Hence, transfer of these CRISPR-Cas systems will almost always occur in a RecG background. These results suggest that our analysis is capable of uncovering previous negative and positive interactions between systems, except when these are nearly ubiquitous.

### (c) Untangling the network of interactions

We observed 51 new significant associations beyond the handful previously reported (figure 2). The first striking observation is that the co-occurrence patterns are not necessarily the same in Firmicutes and Proteobacteria for homologous systems. In some cases, co-occurrences significant in one clade are not significant in the other. This may result from lack of statistical power when CRISPR-Cas or DSB-RS are not equally distributed across the clades. For example, there are 527 genomes encoding subtype I-E systems in Proteobacteria and only 36 in Firmicutes, explaining why the latter shows no significant association for this subtype. DSB-RS are also distributed unevenly among phyla explaining why RuvC has many interactions in Firmicutes where its frequency is intermediate but none in Proteobacteria where it is almost ubiquitous (98% of the genomes encode RuvC) (figure 1). Some clades have strong epistatic groups. For example, RecU and RuvC in Firmicutes exhibit an opposite distribution of associations with Cas subtypes. RuvC is positively associated with types I and III systems, but negatively associated with type II-A systems, while RecU presents the opposite pattern. It is important to highlight that significant associations may result from indirect associations with other traits. In particular, some DSB-RS present a complementary distribution (bacteria have one or the other). A direct consequence of this is that association involving a specific DSB-RS will impact co-occurrence patterns with other DSB-RS. For example, most Proteobacteria encode either AddAB or RecBCD. As a consequence, a positive or negative interaction with one of these systems will yield the opposite association with the other, regardless of the existence of an actual molecular interaction.

We found only one case where a CRISPR-Cas system and a DSB-RS system show opposite associations in Firmicutes and Proteobacteria. AddAB is negatively associated with subtype I-B in Firmicutes, but this interaction is positive in Proteobacteria. As described above, a significant association does not necessarily reflect a mechanistic interaction, which could explain this incongruence. In Proteobacteria, RecBCD is negatively associated with this Cas system which could explain why we detect a positive association with AddAB. Experimental work will be necessary to untangle these complex cases.

**Figure 3.**
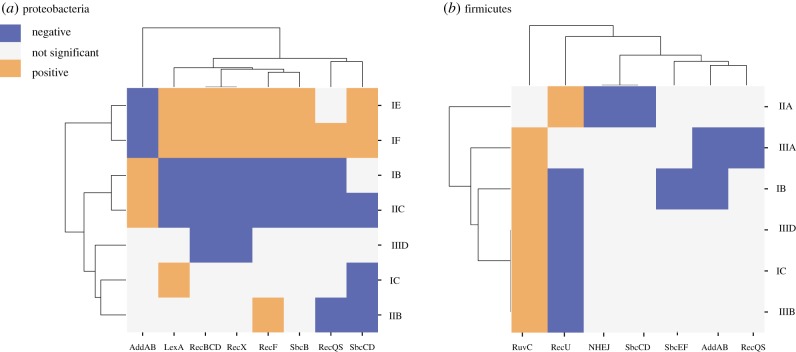
Hierarchical clustering of CRISPR-Cas systems by their associations with components of DSB-RS. Each square corresponds to the association between a CRISPR-Cas system on the *y*-axis and a DNA repair pathway in the *x*-axis. Grey represents no significant association, blue represents a negative association and orange a positive one (Fisher exact test, *p* < 0.05, median of a 100 likelihood ratio tests less than 0.01). Only systems present in more than 1% and in less than 99% of the total number of genomes in the clade and presented at least one significant associations are represented. Associations in (*a*) Proteobacteria and (*b*) in Firmicutes.

There are also cases where systems frequently co-occur. In Proteobacteria, some DSB-RS components—RecBCD, SbcCD, SbcB and LexA—tend to be encoded in the same genomes and thus show similar patterns of co-occurrence with CRISPR-Cas systems. To facilitate the analysis of these evolutionary associations, we performed a hierarchical clustering of the matrix of associations between CRISPR-Cas systems and DSB-RS (figure 3). This analysis clustered subtype I-E and I-F systems in Proteobacteria, which fits previous observations that these two systems are very similar in terms of molecular mechanisms [1]. However, it also revealed unexpected clusters, especially the one grouping together type I-B and II-C in Proteobacteria. These subtypes belong to different types of CRISPR-Cas systems and differ widely in their repertoire of Cas proteins. It is tempting to suggest that either this reflects a common requirement for a given DSB-RS, in this case AddAB, that is positively associated with all these systems, or a negative epistatic interaction of these Cas subtypes with subtype I-E and subtype I-F, which are rare in genomes with AddAB and, at least for subtype I-E, exhibit positive interactions with RecBCD. One surprising observation in this clustering is the opposite pattern of associations between subtypes belonging to the same type of CRISPR-Cas systems such as type I-B and type I-E/I-F. While current knowledge about the biology of these subtypes cannot fully explain this observation, one important difference between these CRISPR subtypes is the presence of the Cas4 protein in type I-B and its absence in type I-E/I-F [3]. In a recent study, Cas4 was shown to enhance spacer acquisition in the absence of RecBCD complex [54]. It is thus possible that systems lacking Cas4 such as types I-E/I-F depend more heavily on RecBCD for adaptation. The presence/absence of Cas4 could therefore be a key factor to explain the observed patterns of associations. The hierarchical clustering thus underlines the diversity and subtype specificity of CRISPR-Cas systems and how they are preferably associated with either RecBCD or AddAB.

In Firmicutes, we observe fewer significant interactions. They are dominated by the negative epistatic interaction between RuvC (present in few species) and RecU (present in most species). To better highlight the complex network of these interactions, we plotted on the Firmicutes phylogenetic tree the distribution of the subtype I-B system and the DSB-RS components with which it is significantly associated (electronic supplementary material, figure S4). Subtype I-B systems are rarely associated with SbcEF, which in turn are rarely associated with RuvC, which in turn is mostly present in genomes that lack RecU. Identifying direct molecular interactions in this complex network will require experimental work.

## 4. Conclusion

We observed significant associations between DSB-RS and Cas subtypes, suggesting synergistic and antagonistic interactions between the two. Detailed explanation of some of these interactions at this stage is difficult for several reasons. First, many of the DSB-RS have been very well studied in only one or two species, and it is not always clear how similar these mechanisms are across phyla. This is even more of a problem for Cas subtypes, many of which have yet to be well characterized even in the model organisms. Second, the differences in molecular mechanisms between Cas subtypes and between epistatic groups of DSB-RS (e.g. RecBCD and AddAB) are not well understood. The helicase activity of RecBCD, notably that of RecB, has recently been shown to be involved in the adaptation of type I-E CRISPR-Cas systems [26]. RecBCD differs from AddAB in several respects, including having one less nuclease domain and an additional helicase domain (RecD). Such differences between analogous systems may be key to explain why some clades favour certain combinations of CRISPR-Cas/DSB-RS. Unfortunately, they are hard to characterize *in vivo* because species typically have only one of the epistatic groups. Third, several key components of DSB-RS are nearly ubiquitous in Bacteria, or at least in the two phyla we studied (e.g. RecN), and their association with CRISPR-Cas cannot be tested with our approach. Fourth, some components have activities beyond their role in DSB-RS. For example, RecBCD is not associated with homologous recombination in Actinobacteria or in Buchnera [49,51], and recombination is probably not the main function of RecG [18]. RecF is involved in DSB-RS only in specific genetic backgrounds and is mostly implicated in the repair of single-strand breaks, those produced by type I systems. These can become DSB upon passage of a replication fork. Finally, Cas and DSB-RS are part of a larger whole and some statistical associations between them may result from interactions of these systems with a third partner in the bacterial cell. The observation that our results capture previous experimental results, that DSB-RS are more associated with Cas subtypes than the average bacterial gene and the fact that both types of systems interact with DNA suggests that many of these links directly involve components of DSB-RS.

**Figure 4.**
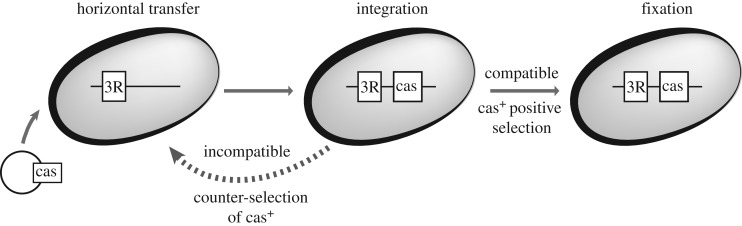
Model of the consequences of the interactions between DSB-RS and CRISPR-Cas systems on CRISPR-Cas system distribution in bacterial genomes. Different subtypes of CRISPR-Cas systems have different compatibilities with resident DSB-RS. When a CRISPR-Cas system is integrated in a bacterial genome, this compatibility will affect its probability of fixation.

Given the number of significant associations between CRISPR-Cas and DNA repair systems, we propose a scenario for the impact of these associations on the distribution of CRISPR-Cas systems (figure 4). CRISPR-Cas systems are subject to frequent horizontal gene transfer. When they are introduced in a novel bacterium, their effect on fitness will depend on a number of factors, such as their expression, their production cost, the impact of phage predation in population dynamics and their role on affecting it, and the presence of other defence systems. We propose that it will also depend on the genetic background, especially regarding the repertoire of the existing DSB-RS functions. Diverse bacteria encode different functions with possibly different degrees of compatibility to specific CRISPR-Cas systems. On one extreme, the CRISPR-Cas system may depend on the existence of a DSB-RS to be fully functional. This seems to underly the positive association of Cas subtype I-E and RecBCD [25]. On the other extreme, there may be an incompatibility between the system and a DNA repair pathway, as we showed previously for subtype II-A and NHEJ [29]. Upon transfer of a new CRISPR-Cas system to a bacterium, the interactions with the existing DSB-RS will contribute to the overall change in fitness resulting from the acquisition of the system, thus driving its loss or fixation in the lineage. Proteins implicated in DSB-RS are under strong purifying selection and we suspect that such incompatibilities will most often lead to the loss of the CRISPR-Cas system and conservation of the extant DSB-RS. As a consequence of this process, bacteria with different DSB-RS will end up encoding different CRISPR-Cas systems. Hence, our results may contribute to explain the scattered distribution and the diversity of these immune systems in bacteria.

Beyond informing what shapes the distribution of CRISPR-Cas systems in bacterial genomes, the interactions reported in this study could lead to new insights into the molecular biology of this defence system. Positive and negative associations with DSB-RS hint at direct molecular interactions and could enlighten novel aspects of the mechanisms behind CRISPR-Cas function. Moreover, the interactions between CRISPR-Cas systems and DSB-RS are at the heart of CRISPR-based technologies for genome editing and for antimicrobial therapy [55,56]. Efficient repair is usually sought for applications regarding genome editing. By contrast, the use of CRISPR-based antimicrobials relies on the impossibility for bacteria to repair efficiently DNA damage introduced by Cas nucleases. A better understanding of the interactions between the two mechanisms in bacteria should thus provide information to improve these technologies.

The major function of DSB-RS is DNA repair. Yet, these systems also play a key role in genetic exchanges between bacteria. On the one hand, they allow the recombination of foreign DNA with homologous regions in the host genome [57]. On the other hand, RecBCD, and presumably AddAB, are powerful exonucleases that may use Chi sites to distinguish self from non-self [58] and protect the cell from invading mobile elements. The latter are known to have developed adequate means of defence. For example, lambdoid phages encoding RecBCD inhibitors lack Chi sites in their genomes, whereas the ones lacking inhibitors encode Chi sites, presumably to subvert self from non-self-discrimination by the bacterial DSB-RS [59]. This implies that molecular interactions between CRISPR-Cas systems and DSB-RS can have multiple consequences from the point of view of bacteria evolvability beyond the well-known effect of CRISPR-Cas systems in protecting the cell from MGEs. Synergistic interactions, such as homologous recombination through the RecBCD pathway allowing the integration of DNA arising by generalized transduction in a CRISPR-Cas genetic background, might lead to increased rates of transfer for bacterial DNA while reducing the ability of MGEs to infect bacterial cells. Antagonistic interactions, such as mechanistic incompatibilities between NHEJ and certain CRISPR-Cas systems, will lead to a reduced repertoire of defence systems that may facilitate infection by MGEs. If the acquisition of a Cas system changes the efficiency of DSB-RS, this will thus affect the rate and type of flow of genetic information in the community. It is therefore conceivable that interactions between DSB-RS and CRISPR-Cas systems affect the rates of gene exchanges in multiple ways.

## Supplementary Material

Supplementary Materials

## Supplementary Material

Supplementary Table 1

## Supplementary Material

Macsy_finder_DNA_repair.zip

## Data accessibility

The datasets generated and analysed during the current study are available from the corresponding author on reasonable request. Macsyfinder definitions, HMM profiles, and detection of CRISPR-Cas systems and DNA repair pathways datasets used in this study are available as electronic supplementary material.
