# Supplementary material for: A matter of background: DNA repair pathways as a possible cause for the sparse distribution of CRISPR-Cas systems in bacteria
Source: Philos Trans R Soc Lond B Biol Sci. 2019 Mar 25;374(1772):20180088. doi: 10.1098/rstb.2018.0088 (PMC6452273; doi:10.1098/rstb.2018.0088)
Supplement: Supplementary Materials [file rstb20180088supp1.pdf]

Supplementary Material for:

A matter of background: DNA repair pathways as a cause for the sparse distribution of CRISPR-Cas systems in bacteria

Aude Bernheim[1,2,3,4,*], David Bikard[3], Marie Touchon[1,2], Eduardo PC Rocha[1,2,*]

[1] Microbial Evolutionary Genomics, Institut Pasteur, 25-28 rue Dr Roux, Paris, 75015, France
[2] CNRS, UMR3525, 25-28 rue Dr. Roux, Paris, 75015, France
[3] Synthetic Biology Group, Institut Pasteur, 25-28 rue Dr. Roux, Paris, 75015, France
[4] AgroParisTech, F-75005 Paris, France

Supplementary files

The file Macsy_finder_DNA_repair.zip attached with the submission contains the HMM profiles and MacSyFinder definitions necessary to search for the DSB-RS in genomes.

Supplementary tables

Supplementary Table 1: Matrix of presence or absence of CRISPR-Cas systems and DNA repair pathways in bacterial genomes.
Excel file attached to the submission.

Supplementary Table 2 : MacSyFinder definitions for the detection of DNA repair pathways. Protein profiles built specifically for this work are labelled "Custom".

ID	Minimum number of proteins required	Proteins	Profiles	Comments
AddAB	2	AddA	Custom (AddA_generic, AddA_epsilon)	The three definitions of AddAB, AdnAB and RecBCD were ran together so that for a defined system it was classified as one of these three, to limit false detection. The parameter of the coverage of the profile – the fraction of the protein profile covered in the alignment with the protein sequence - was tuned to 0.65 instead of 0.5 to increase specificity. Detection of AdnAB only requires one of the proteins to be present as in 21% of the genomes encoding AdnA or AdnB, only one was detected (Fig S1)
		AddB	Custom (AddB_generic, AddB_epsilon)	
AdnAB	1	AdnA	Custom (AdnA)	
		AdnB	Custom (AdnB)	
RecBCD	2	RecB	TIGR00609	
		RecC	TIGR01450	
		RecD	TIGR01447, TIGR01448	
LexA	1	LexA	TIGR00498	
NHEJ	1	Ku*	TIGR02772	The detection relies solely on Ku detection as other ligases than LigD can perform ligation steps[1]. Furthermore, there are two different types of LigD[2]. 74% of the genomes encoding Ku also encoded LigD
		LigD	TIGR02777, TIGR02778, TIGR02779	
RecA	1	RecA	TIGR02012	
RecF	1	RecF	TIGR00611	
RecG	1	RecG	TIGR00643	
RecJ	1	RecJ	TIGR00644	
RecN	1	RecN	TIGR00634	
RecOR	1	RecO	TIGR00613	Even if one single gene is necessary to state that the pair is present in genomes, we observed that 81% of the genomes had both, 18% had only RecR and 0.5% had only RecO (Fig S1).
		RecR	TIGR00615	
RecQS	1	RecQ	TIGR00614	
RecU	1	RecU	TIGR00648	
RecX	1	RecX	PF02631.13	
RuvAB	2	RuvA	TIGR00084	
		RuvB	TIGR00635	
RuvC	1	RuvC	TIGR00228	
SbcB	1	SbcB	Custom (SbcB)	
SbcCD	1	SbcC	TIGR00618	Detection of SbcCD only requires one of the proteins to be present as in 62% of the genomes encoding SbcC or SbcD, only one was detected (Fig S1)
		SbcD	TIGR00619	
SbcE	1	SbcE	Custom (SbcE)	

Supplementary Table 3: Species lacking RecA (and pseudogenes that may have encoded RecA).

Arsenophonus symbiont of *Lipoptena fortisetosa* strain CB
Aster yellows witches'-broom phytoplasma AYWB
Blochmannia endosymbiont of *Polyrhachis* (Hedomyrma) *turneri* strain 675
Buchnera aphidicola str. Sg (Schizaphis graminum)
Candidatus *Blochmannia pennsylvanicus* str. BPEN
Blochmannia floridanus
Candidatus *Blochmannia vafer* str. BVAF
Candidatus *Blochmannia chromaiodes* str. 640
Candidatus *Endolissoclinum faulkneri* L2
Candidatus *Phytoplasma australiense*
Candidatus *Portiera aleyrodidarum* BT-B
Candidatus *Portiera aleyrodidarum* MED (*Bemisia tabaci*)
Candidatus *Profftella armatura*
Candidatus *Ruthia magnifica*
Candidatus *Vesicomyosocius okutanii*
Endosymbiont of *Llaveia axin*
Secondary endosymbiont of *Ctenarytaina eucalypti*
Secondary endosymbiont of *Heteropsylla cubana*
Spiroplasma kunkelii CR2-3x

Supplementary Figures

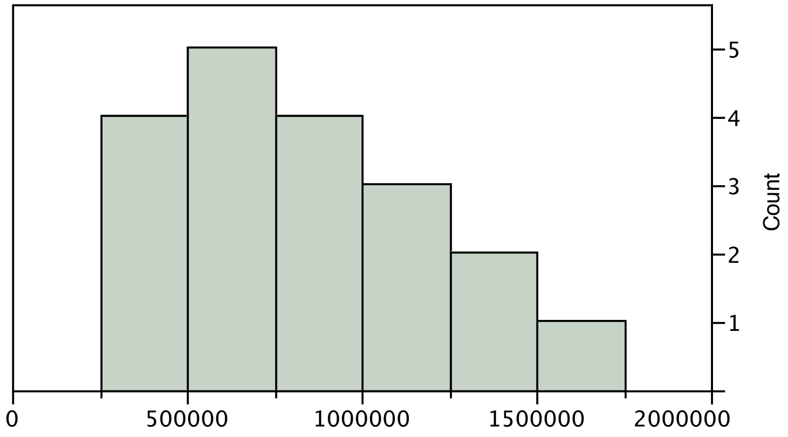

Supplementary figure 1: Histogram of the distribution of sizes (nt) of the genomes of the 20 species lacking RecA.

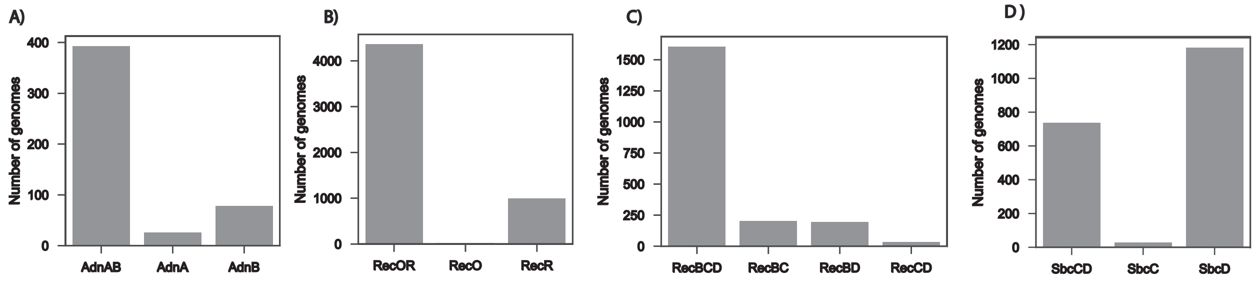

Supplementary figure 2 : Presence of RecOR, RecBCD, AdnAB and SbcCD in bacterial genomes. The columns with the complex (e.g., RecOR) indicate the presence of genes for both proteins, whereas the others indicate the presence of only one of the components (resp. RecO or RecR).

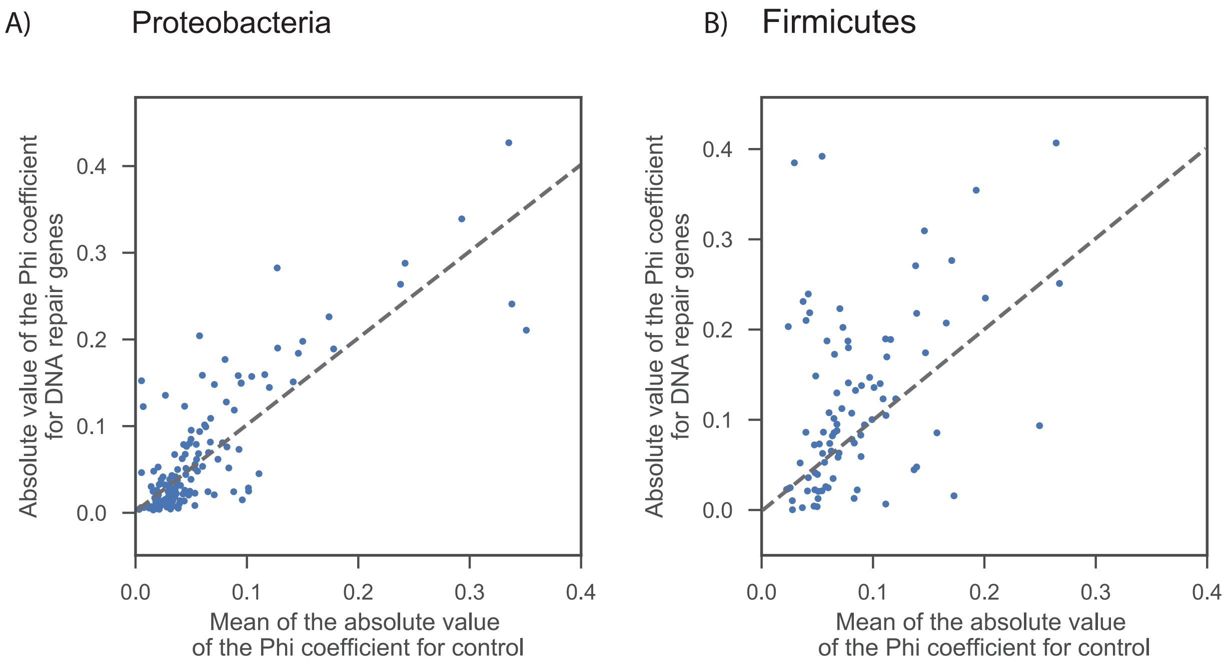

Supplementary figure 3 : Associations of CRISPR-Cas systems with DSB-RS when compared to other cellular functions present at similar frequencies in the phyla.

Each point represents one association between a DSB-RS component and a Cas subtype. The y axis corresponds to the absolute value of the Phi coefficient of the contingency table of the 2 systems. The x axis represents the mean of the absolute value of the Phi coefficient of the 10 contingency tables of the Cas subtypes with 10 genes of similar frequency as the corresponding DNA repair pathway. The dashed line indicates identity, points above the line indicate that correlation is higher for DSB-RS than for the other functions.
